# A visual bias for falling objects

**DOI:** 10.1177/03010066241228681

**Published:** 2024-02-02

**Authors:** Mai Huong Phan, Björn Jörges, Laurence R. Harris, Frederick A. A. Kingdom

**Affiliations:** 7991 York University, Canada; 7991 York University, Canada; 7991 York University, Canada; McGill University, Canada

**Keywords:** motion direction, acceleration, deceleration, gravity, bias, sensitivity

## Abstract

Aristotle believed that objects fell at a constant velocity. However, Galileo Galilei showed that when an object falls, gravity causes it to accelerate. Regardless, Aristotle's claim raises the possibility that people's visual perception of falling motion might be biased away from acceleration towards constant velocity. We tested this idea by requiring participants to judge whether a ball moving in a simulated naturalistic setting appeared to accelerate or decelerate as a function of its motion direction and the amount of acceleration/deceleration. We found that the point of subjective constant velocity (PSCV) differed between up and down but not between left and right motion directions. The PSCV difference between up and down indicated that more acceleration was needed for a downward-falling object to appear at constant velocity than for an upward “falling” object. We found no significant differences in sensitivity to acceleration for the different motion directions. Generalized linear mixed modeling determined that participants relied predominantly on acceleration when making these judgments. Our results support the idea that Aristotle's belief may in part be due to a bias that reduces the perceived magnitude of acceleration for falling objects, a bias not revealed in previous studies of the perception of visual motion.

Aristotle believed that objects fell at constant speed (Hardcastle, 1914; Nature editorial, 1946). A notable empiricist, Aristotle probably arrived at this conclusion in part from his observations of falling objects. Perhaps, then, falling objects do not appear to accelerate as much as physics tells us they do (Galilei, 1638). Our visual world is replete with examples of how continuous exposure to a visual pattern biases our subsequent perception of that pattern. Continuous exposure to falling objects might recalibrate our perception away from acceleration towards that of constant velocity.

Consider an object with a motion profile that varies continuously from deceleration through constant velocity to acceleration. Any perceptual bias will tend to shift the point at which that object is perceived to be moving at constant velocity. We refer to that point as the “point of subjective constant velocity,” or PSCV. If an object appears to accelerate less when moving downwards than when it is moving in other directions, then this would reveal itself as a shift of the downwards PSCV towards the acceleration end of the continuum. Similarly, for upwards-moving objects that in our everyday experience tend to decelerate, the bias would shift the PSCV towards the deceleration end of the continuum. Thus, we hypothesize that the PSCV for downwards-moving objects will be biased towards the acceleration end of the continuum relative to the PCSV for upwards-moving objects. Since leftwards and rightwards moving objects would be expected to accelerate or decelerate equally, we hypothesize no difference in their PSCVs.

Perceptual *bias* is not the same as perceptual *sensitivity*. Previous studies have measured how sensitive we are to acceleration, specifically how much an object needs to accelerate to be discriminable from one that is moving at a constant speed (Calderone & Kaiser, 1989; Lee et al., 2019; Mueller & Timney, 2016; Nakayama & Motoyoshi, 2017; Nguyen & van Buren, 2023; Schmerler, 1976; Watamaniuk & Heinen, 2003; Werkhoven et al., 1992). The studies most relevant to the present one are those by Calderone and Kaiser (1989) and Nguyen and van Buren (2023). They measured sensitivity to acceleration as a function of motion direction, with Calderone and Kaiser (1989) finding no difference between Up and Down and Nguyen and van Buren (2023) finding higher sensitivity for Up than Down. None of the above studies however considers whether subjects are biased in their perception of whether something is accelerating or decelerating depending on the direction of motion or whether any such dependency would be perceptual or cognitive in origin (but see Freyd & Finke, 1984; Hubbard, 2020).

The distinction between appearance-based bias and performance-based sensitivity in the context of the present study is illustrated in Figure 1. The figure shows hypothetical psychometric functions of percentage “it's accelerating” responses as a function of acceleration when asked if a moving object was accelerating or decelerating. The blue and orange curves are hypothetical responses to two directions of motion with the same PSCV but different slopes, that is, sensitivities. The blue and red curves are hypothetical responses to two directions of motion with the same sensitivities but different biases, that is, PSCVs. Such a bias shift indicates that for the red motion direction the object appears to accelerate less for a given acceleration compared to the blue motion direction. Our hypothesis predicts such a difference in bias for Down versus Up.

**Figure 1.**
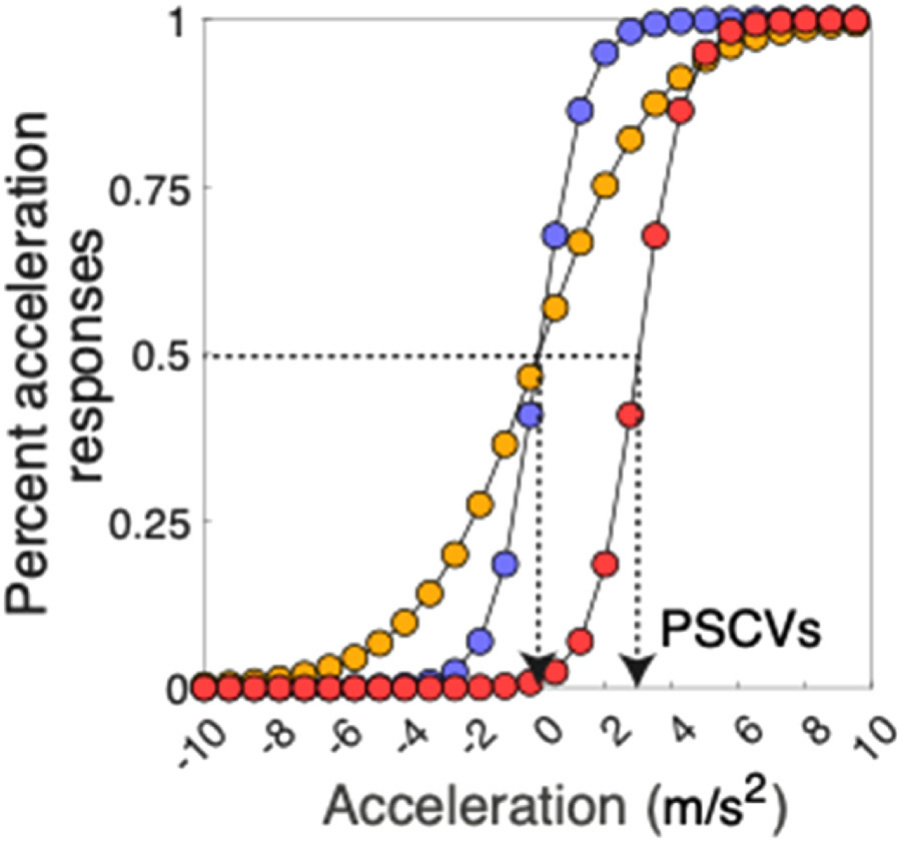
Bias versus sensitivity. Hypothetical psychometric functions describing the percentage of “acceleration” responses as a function of acceleration for three motion directions given by the blue, orange and red curves. Negative values on the abscissa are decelerations. PSCV = point of subjective constant velocity. The difference between the blue and yellow curves is in terms of their slopes indicating a change in sensitivity. The difference between the blue and red curves is in terms of their positions along the abscissa, indicating a change in bias. The colour version is available online.

An issue arises as to which aspect of the motion observers might use to make their decision as to whether the ball was accelerating or decelerating. For a moving object, duration, distance, acceleration, and initial velocity all co-vary. Here we keep duration and distance fixed. In order to do this, initial velocity and acceleration need to co-vary. The question therefore arises as to which cue participants might rely upon to make their judgments as to whether the ball was accelerating or decelerating. To confirm that participants were depending more on the acceleration of the ball than its initial velocity, we used generalized mixed modeling. A brief account of the present study has been given elsewhere (Phan et al., 2022).

## Methods

### Participants

The study was conducted online using Pavlovia (https://opensciencetools.org) with 182 and 158 students from York University taking part in the two parts of the experiment. Participants volunteered for the experiment in exchange for course credit. All participants reported having normal or corrected-to-normal vision and provided written informed consent. The study was approved by the York University ethics board.

### Stimuli

**Figure 2.**
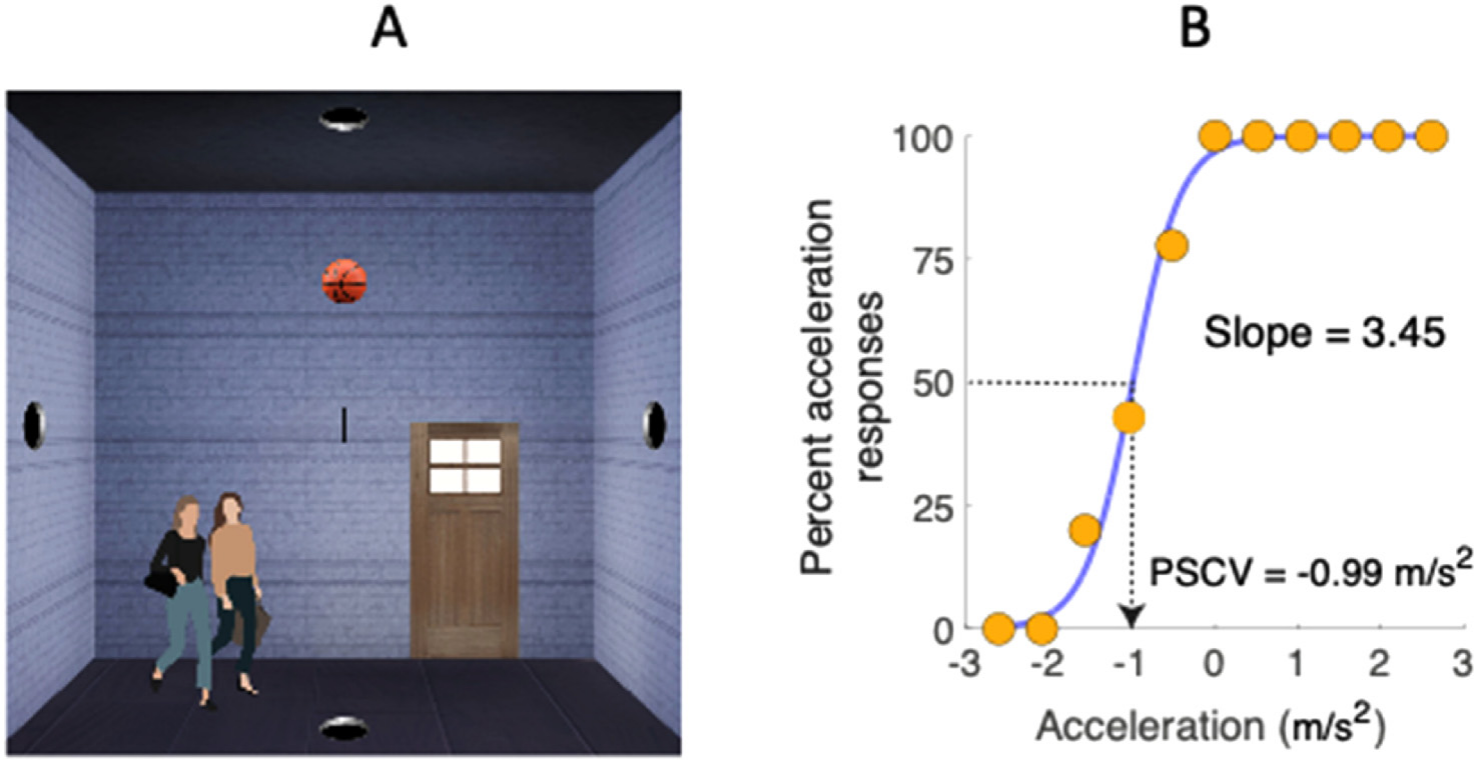
The left panel shows the stimulus display, and the right panel shows a typical psychometric function. Note the fixation cross at the center of the screen and the people and door to provide the scale for the 4 m square room. In the graph on the right, orange circles represent the data points, the blue line is a fitted Logistic function. Other details are as in Figure 1.

Participants judged whether a basketball moving in one of the cardinal directions was accelerating or decelerating across a room which provided clear indications as to its scale (Figure 2, left). The display was shown on their computer screens at home which they were asked to view at approximately 57 cm while maintaining fixation on a cross at the center of the screen. On each trial, a basketball moved across the screen from left to right, right to left, top to bottom, or bottom to top, in a random order. The travel distance was simulated at 4 m, scaled using the sizes of several reference objects such as a door, people, and the basketball itself, as shown in Figure 2a. The ball appeared through one of the holes in the room at an initial velocity chosen such that whether it accelerated or decelerated, the time it took to cross the room was fixed at one of two randomly chosen transit times to eliminate transit time as a cue for whether the ball was accelerating or decelerating. The experiment was run using two groups of participants, one using transit times of 1 and 1.5 s (*n* = 182), the other transit times of 1.25 and 1.75 s (*n* = 158). For each transit time, there were 11 equally spaced acceleration values between  ± 8 m.s^−2^ for a transit time of 1 s,  ± 5.1 m.s^−2^ for a transit time of 1.25 s,  ± 3.56 m.s^−2^ for a transit time of 1.5 s, and ± 2.61 m.s^−2^ for a transit time of 1.75 s. The number of times each acceleration/deceleration value was presented was determined according to an approximate Gaussian distribution, with high acceleration/deceleration values presented less often than low acceleration/deceleration values. This feature maximized the number of values around the PSCV and kept the data collection time to a minimum.

### Procedure

At the start of the experiment the participant was given instructions and conducted 10 practice trials with relatively high levels of acceleration and deceleration in order to become familiar with the task. Feedback (a green tick for correct and a red cross for incorrect) was provided for the practice trials but no feedback was provided during the main experiment as must be the case for an appearance-based experiment measuring bias. On each trial, once the ball had crossed the room, the participant responded “accelerating” or “decelerating” by pressing the left and right arrow keys. For half the participants left meant accelerating and for half of them right meant accelerating. Written instructions about which keys were to be used were given at the start of the experiment. There were 124 trials in total (for both groups) and the experiment took around 20 min to complete.

### Analysis

For each participant and condition, the percentage of times “accelerating” was chosen (*pA*) and was plotted against the deceleration or acceleration value *x*, and the resulting psychometric function was fitted with a Logistic function with weightings applied to each point according to the number of times each value of acceleration had been presented. For this purpose, we used the function PAL_PFML_Fit in the Palamedes toolbox (Prins & Kingdom, 2016).

(1) $$pA=100/[1+exp^{-b(x-a)}]$$

where *pA* is the percentage of acceleration responses, *a* is the estimate of the PSCV and *b* the slope of the function. Outliers were removed using the criteria that the absolute value of the PSCV had to be within ± 20 m.s^−2^ and the slope had to be less than 10 m.s^−2^. Participants’ data which did not satisfy these criteria for *both* motion directions and for *both* transit times were discarded.

Because of the confound between the initial velocity and the acceleration of the ball, we ran a generalized linear mixed model (see Appendix B) to assess which cue participants were relying upon most when making their judgments.

## Results

**Figure 3.**
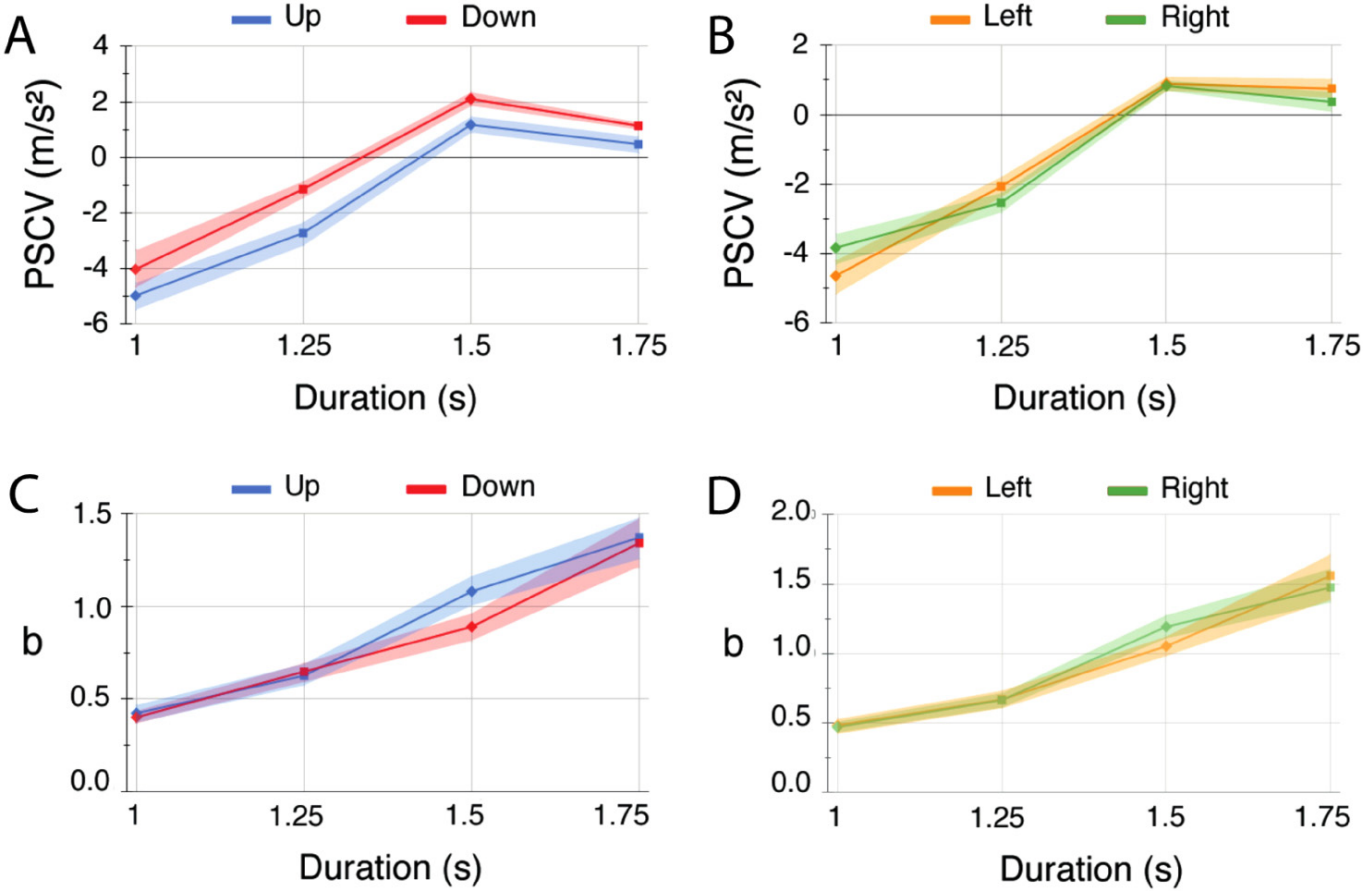
Mean PSCVs and psychometric function slopes b as a function of transit time and motion direction. A = Up/Down PSCVs; B = Left/Right PSCVs; C = Up/Down slopes; D = Left/Right slopes. Shaded areas are standard errors of the mean.

Figure 3 shows PSCVs (A and B) and psychometric function slopes (C and D) for Up versus Down (A and C) and Left versus Right (B and D). The figure shows that PSCVs varied with the ball's transit time, with faster transit times evoking more “acceleration” responses, pushing the PSCV towards the deceleration end of the deceleration–acceleration continuum. This is indicative of a bias for “fast = accelerating.” However, the bias we are primarily interested in here is that between Down and Up and for this comparison it is the relative positions of the PSCVs for Down and Up that is important. As our statistical analyses (below) confirm, for all transit times, Figure 3a shows that PSCVs for Down are relatively displaced towards acceleration compared to those for Up. No such difference can be seen for Left versus Right (Figure 3b).

As discussed earlier, biases are different from sensitivities. But could our results nevertheless be a byproduct of a sensitivity difference between Down and Up? Sensitivity is given by the slope parameter *b* in the fitted psychometric functions. As we confirm statistically (below), Figure 3c and d shows no systematic differences in sensitivity between Down and Up or between Left and Right. Means and standard errors shown in Figure 3 are provided in Appendix A.

We ran separate within-participant ANOVAs for the two sets of participants, one set who performed the experiment for the 1.0 and 1.5 s transit times, the other for the 1.25 and 1.75 s transit times. Separate ANOVAs were conducted for Down-versus-Up and Left-versus-Right in order to obtain a direct comparison between the two members of each motion direction orientation.

The ANOVA results are shown in Table 1. We adopted the *p *< .025 rather than *p *< .05 criterion for significance as a Bonferroni correction for performing statistical analyses on two separate transit times.

**Table 1.** PSCV and slope *p* values for significance for direction of motion differences shown in Figure 3, using the ANOVAs described in the text.

		Transit time, s	
		1.0, 1.5	1.25, 1.75
PSCV comparisons	Down vs. Up	F(1,58)* *= 9.84; *p = *.0027 **	F(1,57) = 23.3; *p *< .0001 ***
	*N*	59	58
	Left vs. Right	F(1,56) = 3.52; *p *= .07 ns	F(1,60) = 22.5; *p *= .12 ns
	*N*	57	61
Slope comparisons	Down vs. Up	F(1,58) = 4.78; *p *= .033 ns	F(1,57) = 0.0077; *p *= .931
	*N*	59	58
	Left vs. Right	F(1,56) = 3.167; *p *= .081 ns	F(1,60) = 0.37; *p *= .55 ns
	*N*	57	61

*N* = number of participants; ns = non-significant at the *p* < .025 criterion. The *p*-values for differences in transit time are not shown but were all significant.

Because of the confound between the initial velocity and the acceleration of the ball (see Table A2), we ran a generalized linear mixed model (see Appendix B). This indicated that although both variables explained a significant amount of the variability, all model fit criteria favored the acceleration-only model when compared to an initial velocity-only model. That is, while participants did rely on both cues, they relied more on acceleration than on the initial velocity.

## Discussion

We have revealed a novel bias difference between the perception of upwards and downwards motion in which downwards motion requires more acceleration than upwards motion to be judged as moving at constant velocity.

As mentioned in the introduction, a recent study by Nguyen and van Buren (2023) found greater sensitivity to Up compared to Down, which would predict higher values of the slope parameter *b* in our study. Our ANOVAs however revealed no significant differences in slopes either between Up and Down or between Left and Right, although, in keeping with Nguyen & van Buren, there is a visibly higher average slope for Up compared to Down with the 1.5 s transit time (see Figure 3c). There are many differences in the experimental methods employed by Nguyen and van Buren (2023) and our study that could potentially account for the differences in sensitivities measured. Nguyen and van Buren (2023) presented only acceleration and constant speed trials, the former with just one value of acceleration, and participants were required to indicate “acceleration” or “constant speed” on each trial. In our study we presented a range of accelerations and decelerations with the participants’ task being to judge “accelerating” or “decelerating” on each trial. Perhaps the fact that in Nguyen & van Buren's study half the trials were constant speed and the other half just one value of acceleration served to sharpen participants’ sensitivities.

Nguyen and van Buren (2023) argue that the higher sensitivity they observed for Up versus Down was due to a visual prioritization of speed changes in the opposite direction to gravity, because such speed changes are indicative of the presence of animate forces. It does not follow however that greater sensitivity to Up than Down speed changes necessarily leads one to expect the bias differences in Up and Down PSCVs found here.

One might suppose that participants would have responded “accelerating” relatively more often than “decelerating” to Down compared to Up on the grounds that they expected downwards-moving objects to accelerate and upwards-moving objects to decelerate. Indeed, much evidence supports such an expectation bias (reviewed by Nguyen & van Buren, 2023). However, such an expectation bias would predict the opposite of what we found; an expectation bias would predict that to compensate for the bias, the Down PSCVs would lie to the left of the Up PSCVs on the deceleration-to-acceleration axis. Therefore, it appears that the perceptual bias revealed in our study overrides any cognitively driven expectation bias. Despite participants being explicitly instructed not to track the ball, an asymmetry in eye tracking (Ke et al., 2013) could have inadvertently influenced the results. Although we did not track eye movements in this study, future investigations could investigate such a possibility.

To focus our observer's attention on the movement of the ball, our experiments used a fixed distance and a fixed time of transition. This meant that although participants could not use distance or time to deduce the motion of the ball, there was a correlation between both the end velocity and the initial velocity of the ball and its acceleration. There is a suggestion in the literature that people are not very sensitive to visual acceleration (Watamaniuk & Heinen, 2003) and that they might be deducing the answer to the question “was it accelerating or decelerating?” from a comparison of the start or end velocities to the average velocity over the course of the experiment. Our claim does not require a direct perception of acceleration – we remain neutral for the moment as to whether humans can or cannot detect visual acceleration directly – and instead claim, as stated in the title, that there is a visual bias for falling objects, one that is not present for sideways moving objects. However, the results from the general mixed modeling indicate that while participants did rely on both cues, they relied significantly more on acceleration than on the initial velocity.

We suggest that the results of the present study reflect a perceptual bias that might be due to the regular exposure of objects accelerating downwards and decelerating upwards. This experience may have led to the internalization of a gravity prior (Jörges & López-Moliner, 2017, 2020) although this could actually be innate (Bliss et al., 2023). This internalized assumption would then underlie the bias we saw. For Left versus Right visual motion there is no regularly experienced differential amounts of acceleration or deceleration, hence we would not expect, nor do we find, a similar bias. In the future, more studies should be conducted to see whether this effect is present in other contexts—such as if participants are tested in the microgravity of space or in a simulated underwater environment.

### Conclusion

Aristotle believed that falling objects fell at a constant velocity. Our results support the idea that his belief may in part be due to a bias that acts to reduce the perceived magnitude of acceleration for falling objects, a bias that to our knowledge has never been revealed in previous studies of visual motion.

## Appendix A: Means and standard errors of the results shown in Figure 3.

See Table A1.

**Table A1.** Mean PSCVs (m.s^−2^) with standard errors (SEs) and mean slopes with SEs for the data points shown in Figure 3.

			Transit time, s			
	Direction	Statistic	1.0	1.25	1.5	1.75
PSCVs	Down	Mean ± SE	−4.04 ± 0.57	−1.15 ± 0.36	2.10 ± 0.29	1.13 ± 0.15
	Up	Mean ± SE	−4.99 ± 0.49	−2.73 ± 0.41	1.17 ± 0.33	0.47 ± 0.31
	Left	Mean ± SE	−4.65 ± 0.47	−2.07 ± 0.29	0.89 ± 0.22	0.75 ± 0.27
	Right	Mean ± SE	−3.84 ± 0.44	−2.54 ± 0.30	0.83 ± 0.16	0.37 ± 0.39
Slopes	Down	Mean ± SE	0.401 ± 0.030	0.648 ± 0.057	0.89 ± 0.084	1.343 ± 0.133
	Up	Mean ± SE	0.423 ± 0.042	0.626 ± 0.061	1.081 ± 0.084	1.373 ± 0.112
	Left	Mean ± SE	0.481 ± 0.04	0.668 ± 0.058	1.052 ± 0.077	1.560 ± 0.162
	Right	Mean ± SE	0.469 ± 0.031	0.663 ± 0.054	1.192 ± 0.086	1.475 ± 0.124

## Appendix B: Additional analyses

In order to ascertain to what extent participants used the acceleration of the object relative to its initial velocity to make their judgments, we opted for model fit comparisons. For this purpose, we relied on the small amount of variability that was not shared between acceleration and initial target velocities (see Table A2).

**Table A2.** Showing the initial velocities and accelerations necessary to keep durations and distance constant.

Participant group 1				Participant group 2			
initial_vel (m/s)	accel (m/s/s)	duration (s)	repetitions	initial_vel (m/s)	accel (m/s/s)	duration (s)	repetitions
6.4	−5.12	1.25	2	8	−8	1	2
5.76	−4.1	1.25	3	7.2	−6.4	1	3
5.12	−3.07	1.25	5	6.4	−4.8	1	5
4.57	−2.61	1.75	2	5.333	−3.556	1.5	2
4.11	−2.09	1.75	7	5.6	−3.2	1	7
4.48	−2.05	1.25	3	4.8	−2.844	1.5	3
3.66	−1.57	1.75	5	4.267	−2.133	1.5	5
3.2	−1.04	1.75	9	4.8	−1.6	1	9
3.84	−1.02	1.25	7	3.733	−1.422	1.5	7
2.74	−0.52	1.75	9	3.2	−0.711	1.5	9
3.2	0	1.25	10	4	0	1	10
2.29	0	1.75	10	2.667	0	1.5	10
1.83	0.52	1.75	9	2.133	0.711	1.5	9
2.56	1.02	1.25	7	1.6	1.422	1.5	7
1.37	1.04	1.75	9	3.2	1.6	1	9
0.91	1.57	1.75	5	1.067	2.133	1.5	5
1.92	2.05	1.25	3	0.533	2.844	1.5	3
0.46	2.09	1.75	7	2.4	3.2	1	7
0	2.61	1.75	2	0	3.556	1.5	2
1.28	3.07	1.25	5	1.6	4.8	1	5
0.64	4.1	1.25	3	0.8	6.4	1	3
0	5.12	1.25	2	0	8	1	2

While initial velocity and acceleration were correlated with an *R*^2^ of about 0.9, this leaves a non-negligible share of variation that was unique to acceleration and velocity, respectively. And given the large number of participants (and with it, the high statistical power), we could leverage this small part of non-shared variability to determine which of the two sources of information they preferred. This model fit-based process has been used in at least one other study conducted to determine which cues participants used to make judgments about accelerations (Jörges et al., 2018). Following the approach laid out by Moscatelli et al. (2012), we fitted four generalized linear mixed models with logit link functions using the lme4 package for R (R Core Team, 2017). For all models, we set the participants’ response on any given trial as dependent variable, random slopes for the motion direction as well as random intercepts per participant as random effects and the motion direction as fixed effect:

(1)
Response∼Direction+(Direction|Participant)

This was the full structure of the first model (“Baseline Model”). We also fitted a model (“Full Model”) that, additionally, had both initial velocity and acceleration as fixed effects:

(2)
Response∼Acceleration+InitialVelocity+Direction+(Direction|Participant)

We further fitted an acceleration-only model:

(3)
Response∼Acceleration+Direction+(Direction|Participant)

And a velocity-only model:

(4)
Response∼InitialVelocity+Direction+(Direction|Participant)

We then compared the four models using likelihood ratio tests as implemented in the anova() function from Base R to compare their model fits, while penalizing for additional parameters. The likelihood ratio tests showed that all three more complex models (full model, acceleration-only model, velocity-only model) had better model fits than the Baseline model even after accounting for the additional parameters (*p* < .0001). Further, the full model was significantly better than both the acceleration-only model (*p* < .0001) and the velocity-only model (*p* < .0001), indicating that participants relied both on acceleration and velocity information.

Finally, in order to compare the acceleration-only and velocity-only models, a likelihood ratio test was not necessary since both models had the same number of parameters. The Akaike information criterion (AIC) values, where a lower AIC indicates the better model, favored the acceleration model (AIC = 94,554) over the velocity model (AIC = 99,390), with a difference of 4,836. According to Burnham and Anderson (2002, 2004), an AIC difference of just 7 or more is significant. The dominance of the acceleration only model was also confirmed with a Bayesian information criterion analysis (where smaller is also better) with a score of 94,693 for the acceleration only model and 99,529 for the velocity only model. Finally, a log-likelihood analysis (where this time, bigger is better) gave −47,262 for the acceleration-only model and −49,680 for the velocity-only model. Together these analyses constitute strong evidence that, whereas our participants also used velocity information, they relied *more* on acceleration cues to make their judgments.
